# Current Approaches to the Use of Artificial Intelligence for Injury Risk Assessment and Performance Prediction in Team Sports: a Systematic Review

**DOI:** 10.1186/s40798-019-0202-3

**Published:** 2019-07-03

**Authors:** João Gustavo Claudino, Daniel de Oliveira Capanema, Thiago Vieira de Souza, Julio Cerca Serrão, Adriano C. Machado Pereira, George P. Nassis

**Affiliations:** 1 University of São Paulo, School of Physical Education and Sport - Laboratory of Biomechanics, Av. Prof. Mello de Morais, 65 – Cidade Universitária, São Paulo, São Paulo 05508-030 Brazil; 2 Research and Development Department, LOAD CONTROL, Contagem, Minas Gerais Brazil; 3 Computing Department, Federal Center for Technological Education of Minas Gerais, Belo Horizonte, Brazil; 4 Computer Science Department, Federal University of Minas Gerais, Belo Horizonte, Brazil; 5 Department of Sports Science, City Unity College, Athens, Greece; 6 School of Physical Education & Sport Training, Shanghai University of Sport, Qingyuanhuan Rd 650, Yangpu District, Shanghai, 200438 China

**Keywords:** Artificial neural networks, Machine learning, Technology, SportsTech, Innovation, Analytics, Soccer, Basketball, Handball, Volleyball

## Abstract

**Background:**

The application of artificial intelligence (AI) opens an interesting perspective for predicting injury risk and performance in team sports. A better understanding of the techniques of AI employed and of the sports that are using AI is clearly warranted. The purpose of this study is to identify which AI approaches have been applied to investigate sport performance and injury risk and to find out which AI techniques each sport has been using.

**Methods:**

Systematic searches through the PubMed, Scopus, and Web of Science online databases were conducted for articles reporting AI techniques or methods applied to team sports athletes.

**Results:**

Fifty-eight studies were included in the review with 11 AI techniques or methods being applied in 12 team sports. Pooled sample consisted of 6456 participants (97% male, 25 ± 8 years old; 3% female, 21 ± 10 years old) with 76% of them being professional athletes. The AI techniques or methods most frequently used were artificial neural networks, decision tree classifier, support vector machine, and Markov process with good performance metrics for all of them. Soccer, basketball, handball, and volleyball were the team sports with more applications of AI.

**Conclusions:**

The results of this review suggest a prevalent application of AI methods in team sports based on the number of published studies. The current state of development in the area proposes a promising future with regard to AI use in team sports. Further evaluation research based on prospective methods is warranted to establish the predictive performance of specific AI techniques and methods.

**Electronic supplementary material:**

The online version of this article (10.1186/s40798-019-0202-3) contains supplementary material, which is available to authorized users.

## Key Points

- AI techniques or methods currently having been employed for sporting performance prediction are artificial neural network, decision tree classifier, Markov process, and support vector machine in sports such as basketball, soccer, and volleyball.
- For injury risk assessment, the artificial neural network, decision tree classifier, and support vector machine have been used in soccer, basketball, American football, Australian football, and handball.
- Application of AI methods in team sports has the potential to grow further given continued development of the field and implementation of evaluation research in sports practice to establish the predictive performance of each specific technique/method.

## Background

Artificial intelligence (AI) techniques and methods have attracted considerable attention in the information industry and in society as a whole, due to the large amount of data and the imminent need to transform this data into useful knowledge and practical solutions [1–3]. However, the effective use of data in some areas is still under development, as is the case in sports. As in most other areas of society, increasing volume of data has been gathered in all kinds of sports, and automated data analysis became an important and fast developing field. Careful analyses of these large data sets can enhance our knowledge in sport sciences while at the same time assist in the decision-making of the practitioners who work on the optimization of training and competition strategies [4, 5].

Data science has emerged as a strategical area to exploit knowledge in sports science aiming to fill some gaps left by traditional statistical methods. As a hybrid knowledge area, data science is more than the combination of statistics and computer science as it requires training in how to weave statistical and computational techniques into a larger framework, problem by problem, and to address discipline-specific questions [6]. A holistic view of data science requires an understanding of the context of data, appreciating the responsibilities involved in using private and public data, and clearly communicating what a dataset can and cannot tell us about the real world [6], in our case, in the sports world. Based on learning models, the algorithms can be tuned and be optimized in order to produce better results for supporting decisions and provide applied knowledge to athletes and sport professionals. These algorithms are applied as supervised learning (e.g., classification and regression) and unsupervised learning (e.g., clustering). The supervised learning requires input and output data to develop a predictive model whereas the unsupervised learning is based on input data only [7]. Data science is an emerging area in both industry and academic ways, leading to more evidence-based decision-making across many walks of life, including social networking services, streaming services, health care, manufacturing, education, financial modeling, policing, and marketing [8–11]. Also, in our area, linking science and technology to increase sports efficiency has been touted as a path with a promising future. For this to happen, the work directed toward the innovation, introduction, and improvement of processes performed by research and development (R&D) departments in the world’s largest technology companies has been also suggested in the sport field. Because of its fast-moving environment, sport professionals combine data (e.g., physical, technical and tactical) with their expert opinion to inform decisions on the players [12].

However, success in team sport, from the sport science and medicine staff perspective, is to use evidence-based knowledge in an effective manner to develop the decision-making process for injury risk reduction and athletes’ performance optimization [13]. How can the adoption of the research findings and innovations be improved hence improving the compliance of players and coaches/managers with injury prevention and performance enhancement programs? To answer this question, we must accept that players’ needs come first and that players and coaches are the main actors. Therefore, we need to add value to them, offering solutions that really affect their daily lives [13].

One key issue in the sports industry is the possibility to predict injury risk and performance. Historically, the ability of the coaching staff to prescribe training to achieve optimal athletic performance with low risk of injury can be attributed to many years of personal experience. However, modern approaches aiming in adopting scientific methods for the effective development of optimal training programs are warranted [14]. The application of contemporary statistical approaches from AI open an interesting perspective for dealing with injury prevention and for improving the performance models [15, 16].

Therefore, the understanding of the state-of-the-art of AI techniques or methods applied to team sport warrants investigation. With this in mind, the purpose of this study is to give an overview of the current state of the application of AI in team sports. In particular, our review is aiming in answering the following questions: (1) Which AI approaches have been applied to studies investigating injury risk and sport performance in team sports? (2) What are the team sports that have been using AI techniques for predicting injury risk and athletes' performance?

## Methods

### Procedures

The review methodology adopted the Preferred Reporting Items for Systematic Reviews and Meta-Analyses (PRISMA) guidelines [17]. The selection process and data extraction methods were completed by JGC, DOC, and TVS. The quality appraisal was completed by the same authors.

### Search Strategy

Three electronic databases (PubMed, Web of Science, and Scopus) were systematically searched up to May 2018. The command line (“machine learning” OR “predictive modeling” OR “injury prediction” OR “learning algorithms” OR “data mining” OR “naïve bayes” OR “logistic regression” OR “random forest” OR “support vector machine” OR “neural network” OR “deep learning” OR “artificial intelligence” OR “extreme learning machines” OR “data science” OR “knowledge discovery” OR “injury forecasting” OR “injury detection” OR “decision trees” OR “business intelligence”) AND (“team sport” OR “team sports” OR “sport” OR “sports” OR “individual sports” OR “individual sport” OR “athlete” OR “athletes”) AND (“monitoring” OR “load” OR “training load” OR “controlling” OR “control” OR “load control” OR “regulating” OR “regulation” OR “managing” OR “management” OR “improvement” OR “improve” OR “optimizing” OR “optimize” OR “enhance” OR “enhancement” OR “performance” OR “reduce” OR “reducing” OR “decrease” OR “decreasing” OR “injury risk” OR “injury prevention”) was used during the electronic search and applied for the last 5 years to find out the current state of the application of AI in team sports.

### Eligibility Criteria and Selection Process

The first author (JGC) reviewed and identified the titles and abstracts based on the following inclusion criteria with two other authors (DOC and TVS) double checked. If some doubt arose, the fourth author (ACMP) was involved for the final decision. The inclusion criteria were:

1. The study was written in English
2. The study was published as a full-text, original research paper in a peer-reviewed journal
3. Data was reported just for team sport or individual sport athletes and split into distinct groups
4. The participants were competitive athletes (defined as olympic, international, professional, semi-professional, national, youth elite or division I collegiate)
5. The AI techniques or algorithms should be described and tested

### Quality Assessment

The quality of all studies was evaluated using evaluation criteria (Table 1) described by Saw et al. [18]. Scores were allocated based on how well each criterion was met, assuming a maximum possible score of 7 (low risk of bias). Studies with a risk of bias score of 4 or less were considered poor and were excluded. Once the studies to be included were selected, we performed a review on checking reference lists [19] to identify additional peer-reviewed studies.

**Table 1:** Risk of bias assessment criteria

Criteria		Definition	Scoring		
			0	1	2
A	Peer-reviewed	Study published in peer-reviewed journal	No	Yes	–
B	Real-world approach	The approach was performed with real results/data of the athletes	No	Yes	–
C	Population defined	Age, gender, sport, and level was described	No	Partly	Yes
D	Experimental design	Experimental design of the study period was described and replicable	No	Partly	Yes
E	Artificial intelligence	The artificial intelligence approaches/techniques were described	No	Yes	–

### Classifying the Main Research AI Technique or Method

Initially, we considered only the main learning AI technique or method [1–3] for this systematic review. Then, we divided our methodology into two sections, the main method, which corresponds to the main AI technique or method used, and a complete method, which describes all the techniques used in the adopted model by a research paper. This organization aims to facilitate the classification of each paper and compare it with others. Furthermore, we registered the model evaluation metrics used to assess the performance of the technique/method employed. When more than one AI technique or method was evaluated in the same study, we decided to highlight the best model in this paper, that is, the model with the best performance.

## Results

**Fig. 1:**
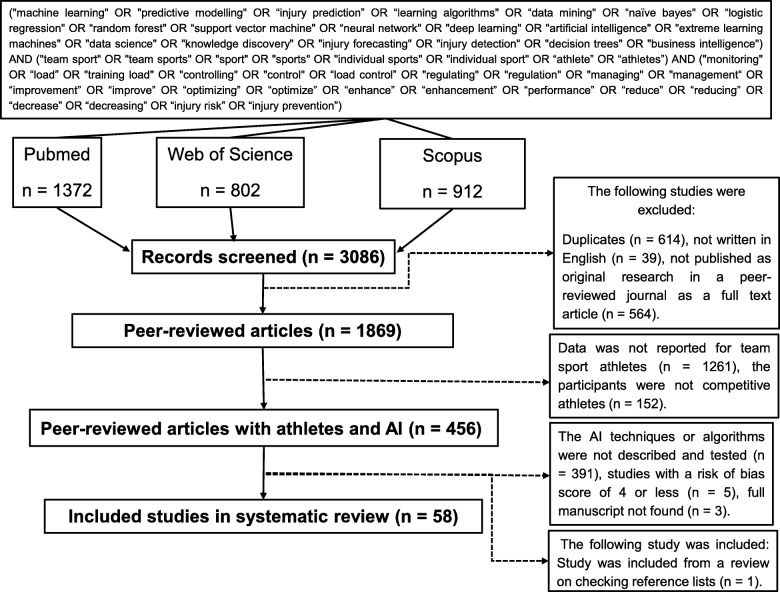
Study selection PRISMA flow diagram

The initial search returned 3086 articles (for details see Fig. 1). After the removal of duplicated articles (*n* = 614), a total of 2472 studies were retained for full-text screening. Following eligibility assessment, 5 studies with a risk of bias score of 4 or less were considered poor and were excluded (for details see Additional file 1: Table S1). The remaining studies were evaluated between 6 and 7 points in terms of quality. We were unable to find the full manuscript for 3 research studies which were excluded from further analysis. During the revision of the reference lists, 1 study found meeting all the criteria and was included in the analysis. Thus, 58 studies in total were included in this systematic review [15, 20–76].

### Characteristics of the Studies

The pooled sample was 6456 participants (i.e., the sum of the subjects of all studies that reported the sample size, i.e., 48% of the studies) with the vast majority being male (97% male, 25 ± 8 years old; 3% female, 21 ± 10 years old). Seventy-six percent of them were professional, 10% youth academy, 9% collegiate, 5% semi-professional and national team U18, and 2% retired professional players. Twenty-six percent of the sample were soccer players, 22% basketball, 10% handball and volleyball, 9% Australian football, 9% baseball, 7% American football, 5% ice hockey, 3% rugby, and 2% each were beach volleyball, cricket, and field hockey players.

### Main Artificial Intelligence (AI) Technique or Method in Team Sports

Eleven AI techniques or methods (Table 2) [1–3] were found in 12 team sports. Approximately two thirds (*n* = 43) of the AI studies were related to sporting performance (74%) whereas 15 studies were related to injury risk (26%). With regard to the injury risk assessment studies, 27% of them were related to training load, 13% to concussion, screening, and, training process/knee injury causes, and 7% each were on ground reaction force pattern, heart defect detection, monitoring based on wearable sensors, psychosocial stress factors, and ulnar collateral ligament reconstruction approaches. With regard to the performance studies, 88% of them were related to technical and tactical analysis, 5% were on physical, technical, and tactical analysis, and 2% each were on match attendance, psychological dynamics of cooperative teamwork, and prediction based on heart rate measures.

**Table 2:** AI techniques or methods descriptions

AI	Description
Absolute shrinkage and selection operator	In statistics and machine learning, least absolute shrinkage and selection operator (LASSO) is a regression analysis method that performs both variable selection and regularization in order to enhance the prediction accuracy and interpretability of the statistical model it produces. It was originally introduced in the geophysics literature in 1986 but was independently rediscovered and popularized by Robert Tibshirani in 1996; he coined the term and provided further insights into the observed performance. LASSO was originally formulated for least squares models. This simple case provides a vast scenario regarding the behavior of the estimator, including its relationship to ridge regression, best subset selection, besides the connections between LASSO coefficient estimates and the so-called soft thresholding. It also reveals that (as standard linear regression) the coefficient estimates need not be unique if covariates are collinear. Although originally defined for least squares, LASSO regularization is easily extended to a wide variety of statistical models including generalized linear models, generalized estimating equations, proportional hazards models, and M-estimators, in a straightforward fashion.
Artificial neural network	An artificial neural network (ANN) is a computational model based on the structure and functions of biological neural networks. Information that flows through the network affects the structure of the ANN because a neural network changes—or learns, in a sense—based on inputs and outputs. ANNs are considered nonlinear statistical data modeling tools whereby the complex relationships between inputs and outputs are modeled or patterns are found.
Bayesian logistic	In statistics, the logistic model (or logit model) is a widely used statistical model that, in its basic form, uses a logistic function to model a binary dependent variable; many more complex extensions exist. In regression analysis, logistic regression (or logit regression) means estimating the parameters of a logistic model; it is a form of binomial regression. Logistic regression is a type of regression analysis used for predicting the outcome of a categorical (a variable that can take on a limited number of categories) dependent variable based on one or more predictor variables. The probabilities describing the possible outcome of a single trial are modeled, as a function of explanatory variables, using a logistic function. Logistic regression measures the relationship between a categorical dependent variable and usually a continuous independent variable (or several), by converting the dependent variable to probability scores. Logistic regression can be binomial or multinomial. In the multinomial type, there exist different models to perform the regression, such as the Bayesian method.
Bayesian networks	Bayesian networks are a type of probabilistic graphical model that uses Bayesian inference for probability computations. Bayesian networks aim to model conditional dependence and, therefore, causation, by representing conditional dependence by edges in a directed graph. Through these relationships, one can efficiently conduct the inference of the random variables in the graph by using factors.
Decision tree classifier	A decision tree classifier (DTC) is a decision support tool that uses a tree-like graph or model of decisions and their possible consequences, including chance event outcomes, resource costs, and utility. It is one way to display an algorithm that only contains conditional control statements. Decision trees are commonly used in operations research, specifically in decision analysis, to help identify a strategy most likely to reach a goal, but are also a popular tool in machine learning. Decision tree learning uses a decision tree (as a predictive model) to go from observations about an item (represented in the branches) to conclusions about the item target value (represented in the leaves). It is one of the predictive modeling approaches used in statistics, data mining, and machine learning. Tree models whereby the target variable can take a discrete set of values are called classification trees; in these tree structures, leaves represent class labels and branches represent conjunctions of features that lead to those class labels.
Fuzzy clustering	Fuzzy clustering is an alternative method to conventional or hard clustering algorithms, which partitions data containing similar subjects. Fuzzy clustering contrasts with hard clustering by its nonlinear nature and discipline of flexibility in grouping massive data. It provides more accurate and close-to-nature solutions for partitions and herein implies more possibilities of solutions for decision-making. In the specific matter of computation, fuzzy clustering has its roots in fuzzy logic and indicates the likelihood or degrees of one data point belonging to more than one group.
*K*-means clustering	*K*-means clustering is a type of unsupervised learning, used when there is unlabeled data (i.e., data without defined categories or groups). The goal of this algorithm is to find groups in the data, with the number of groups represented by the variable *K*. The algorithm works iteratively to assign each data point to one of the *K* groups based on the features provided. Data points are clustered based on feature similarity. The results of the *K*-means clustering algorithm are the centroids of the *K* clusters, which can be used to label new data, and labels for the training data (each data point is assigned to a single cluster). Rather than defining groups before looking at the data, clustering allows finding and analyzing the groups that have formed organically. Each centroid of a cluster is a collection of feature values which define the resulting groups. Examining the centroid feature weights can be used to qualitatively interpret what kind of group each cluster represents.
*K*-nearest neighbor	*K*-nearest neighbor (*k*-NN) is a type of instance-based learning, or lazy learning, in which the function is only approximated locally and all computation is deferred until classification. The *k*-NN algorithm is among the simplest of all machine learning algorithms. Both for classification and regression, a useful technique can be used to assign weight to the contributions of the neighbors, so that the nearer neighbors contribute more to the average than the more distant ones. For example, a common weighting scheme consists in giving each neighbor a weight of 1/*d*, where *d* is the distance to the neighbor. The neighbors are taken from a set of objects for which the class (for *k*-NN classification) or the object property value (for *k*-NN regression) is known. This can be thought of as the training set for the algorithm, although no explicit training step is required. A peculiarity of the *k*-NN algorithm is that it is sensitive to the local structure of the data. The algorithm is not to be confused with *k*-means, another popular machine learning technique.
Markov process	A Markov process is a random process indexed by time, and with the property that the future isindependent of the past. The present. Markov processes, named for Andrei Markov, are among the most important of all random processes. In a sense, they are the stochastic analogs of differential equations and recurrence relations, which are certainly among the most important deterministic processes.
Support vector machine	Support vector machine (SVM) is a discriminative classifier formally defined by a separating hyperplane. In other words, given the labeled training data (supervised learning), the algorithm outputs an optimal hyperplane which categorizes new examples. In a two-dimensional space, this hyperplane is a line dividing a plane into two parts in which each class lies on either side. The learning of the hyperplane in linear SVM is done by transforming the problem using some linear algebra. This is where the kernel plays its role.
Support vector machine + decision tree classifier	The idea of combining SVM and DTC is to provide a hybrid approach, which attempts to embed SVM within a decision tree algorithm as a decision tree pre-pruning method and resulting into a more accurate and efficient hybrid classifier.

**Fig. 2:**
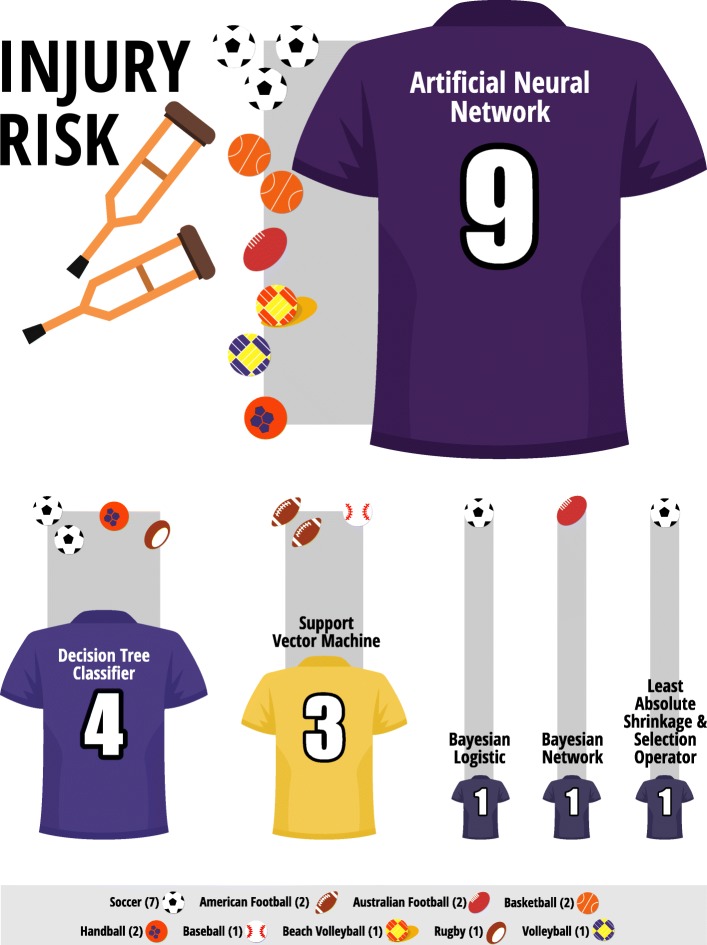
AI for predicting injury risk in various sports

**Fig. 3:**
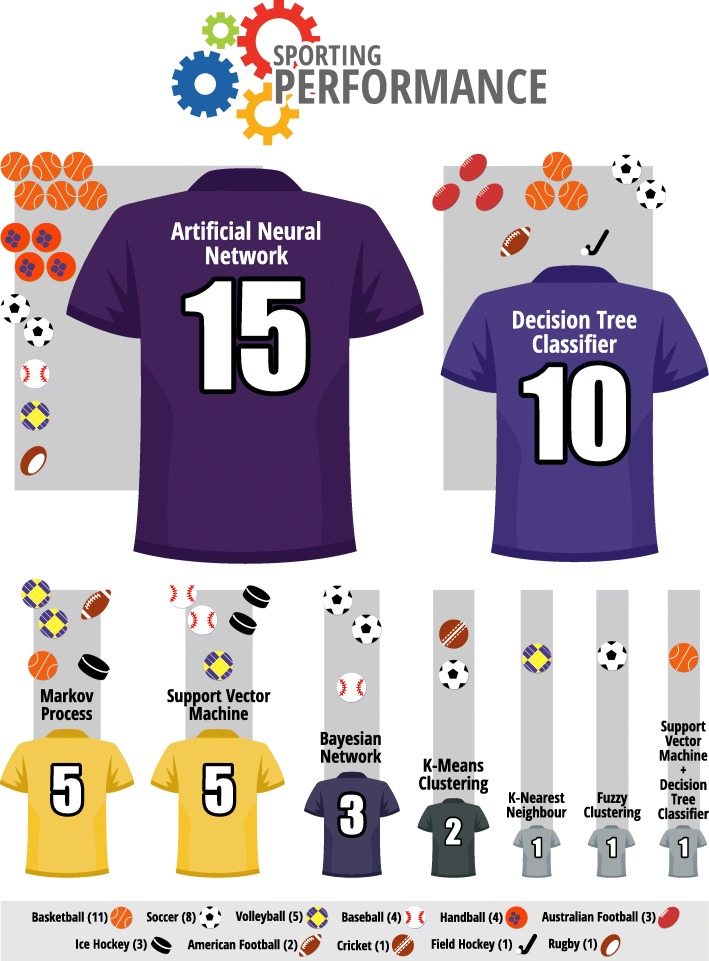
AI for predicting sporting performance in various sports

In the last 5 years, the main AI technique or method used for injury risk assessment and sporting performance prediction was artificial neural network (10% of the injury risk and 26% of the sports performance prediction studies, reporting its use). For injury risk assessment studies, the decision tree classifier and support vector machine (5%) were the next mostly used techniques and methods. In the performance prediction domain, the decision tree classifier (17%), Markov process, and support vector machine (9%) were the most frequently AI techniques/methods used. AI for injury risk assessment was applied to soccer (12% of the studies), basketball, American football, Australian football, and handball (3%) whereas basketball (19%), soccer (14%), and volleyball (9%) were the sports which mostly used performance prediction algorithms. The AI techniques or methods with the best model evaluation metrics were indicated to be applied (for details see Additional file 2: Table S2). Furthermore, 11 studies [22, 25, 26, 29, 37, 48, 49, 52, 53, 55, 69] did not report the evaluation metrics specific for the model. However, the authors of the latter studies recommended the application of AI techniques or methods tested in each manuscript. Only one study did not recommend the use of the tested AI [70] (for details see Additional file 2: Table S2; Figs. 2 and 3). AI techniques or methods had better performance metrics than traditional statistical methods for predicting injury risk and athletes’ performance in 8 studies [31, 35, 38, 54, 57, 67, 70, 71] that made this comparison.

## Discussion

The main purpose of this study is to provide an overview of the current state of the application of AI in assessing the injury risk and predicting performance in team sports athletes. The AI techniques or methods with the greatest potential were artificial neural networks, decision tree classifier, Markov process, and support vector machine.

Lapham and Bartlett [77]’s study was one of the first studies using AI in the analysis of sports performance. In 1995, they demonstrated that the increasing role of computers in the decision-making process, through the use of AI techniques, would be a potentially rewarding future direction for the discipline and already pointed out the artificial neural networks successes in experiments, along with their potential benefits. Another study [78] published over 20 years ago reported the use of AI, with decision tree classifier and Bayesian classification, in the diagnosing of sport injuries. Naive Bayesian classifier with the fuzzy discretization of numerical attributes was superior to other methods and was estimated as the most appropriate for practical use [78]. However, this method is not so popular nowadays, whereas the decision tree classifier has been mostly used so far.

McCall et al. [12] recommended that professional soccer teams included R&D into their daily activities to reduce injuries and to optimize performance. Most of the participants in the studies included in our paper were soccer players (26%), and this could be due to the fact that most of the studies were in soccer. In this particular sport, AI was applied to “training load” [71, 73], “training process/knee injury causes” [63], “heart defect detection” [64], “ground reaction force pattern” [65], “psychosocial stress factors” [69], and “screening” [15]. In summary, the AI techniques or methods applied to predict the injury risk (*n* = 7 studies) in the sample are composed of collegiate (14%) and professional (86%) soccer players and were distributed as following: 43% were on artificial neural network [63–65], 29% on decision tree classifier [15, 71], and 14% on each of Bayesian logistic regression [69] and least absolute shrinkage and selection operator [73] (for details see Additional file 2: Table S2). Regarding the soccer performance domain, AI was applied to “technical and tactical analysis” [30, 36, 44, 45, 48, 50], “match attendance” [31], as well as on “psychological dynamics of cooperative teamwork” [35]. The AI techniques or methods used to predict sporting performance (*n* = 8 studies) in youth academy (13%), semi-professional (13%), and professional (75%) soccer players were as follows: 25% each of artificial neural network [30, 31], Bayesian network [35, 36] and decision tree classifier [44, 45], and 13% each of fuzzy clustering [48] and *K*-means clustering [50]. A great challenge for the future is the application of AI models to integrate all related variables for injury risk assessment and performance prediction.

The second sport in AI application was basketball. Most of the studies on performance were in the “technical and tactical analysis” area [20, 22–25, 41–43, 55, 62], whereas one study was on the “physical, technical, and tactical analysis” [21]. Their focus justifies the recognized importance of physical, technical, and tactical analysis for basketball success [79, 80]. On a separate note, one study described a system for the automatic detection and tracking of the ball trajectory during a free throw based on algorithms [81]. According to the authors of that paper, coaches using this system will be able to monitor the trajectory of the ball and the parameters of the free throw, a fact that will help them correct the technique and hence improve the athletic performance of basketball players [81]. Our analysis showed that the AI techniques or methods used to predict sporting performance (*n* = 11 studies) were with youth academy (9%), semi-professional (9%), and professional (82%) basketball players. The methods reported were as follows: on each artificial neural network (55%) [20–25], on decision tree classifier (27%) [41–43], and 9% each of Markov process [55] and support vector machine + decision tree classifier [62]. For injury risk assessment, the studies were based on “training process/knee injury causes” [66] and “heart defect detection” [64]. The prediction of knee injury risk is essential due to the high costs of medical treatment [82] and to the high rates of medically disqualifying injuries among young athletes [83]. Heart defect detection for basketball players is also highly desired because sudden cardiac death risk among males was estimated at more than 10 times higher than that in the overall athletic population of the National Collegiate Athletic Association (NCAA) Division 1 (i.e., 1 in 5,200 vs. 1 in 53,703 athletes per year) [84, 85]. For injury risk prediction, the most frequently used AI technique or method (*n* = 2 studies) applied in collegiate and professional basketball players was artificial neural network [64, 66].

AI application into handball and volleyball were well reported. In terms of injury risk assessment, ground reaction force patterns is a standard method of investigation in sports medicine and biomechanics [65] that is also related to knee injuries in multidirectional sports, such as handball and volleyball [86, 87]. These injuries may occur during activities, such as jump landing, cutting, and pivoting [86, 87]. Furthermore, injury prediction with AI technique or method based on “screening” [15] was successfully applied to handball players. This approach may assist in identifying the injury risk with higher probability compared to the use of isolated screening tests [88, 89]. The AI techniques or methods (*n* = 2 studies) used to predict injury risk in professional handball (100%) and volleyball (50%) players were artificial neural network [65] and decision tree classifier [15]. For handball performance, all the studies were on the “technical and tactical analysis” area with youth academy based studies using artificial neural network (*n* = 4 studies) [26–29]. For volleyball performance, most of the studies were on the “technical and tactical analysis” area too [33, 52, 53, 61] and only one, with professional players, was on “physical, technical, and tactical analysis” [51]. AI techniques or methods mostly used for performance prediction (*n* = 5 studies) were the Markov process (40%) [52, 53], artificial neural network (20%) [33], *K*-nearest neighbor (20%) [51], and support vector machine (20%) [61]. The areas of physical, technical, and tactical analysis are of high importance to athletic performance in both sports [90, 91], and coaches/players may now be able to use the tools of AI for better decision-making.

Training load management is a concern in Australian football [92–94]. Along this line, AI methods and techniques have been used to predict the risk of injury through the “training load” [67] and “screening” [70] in that sport. Interestingly, there is only one study where the authors did not recommend the supervised learning techniques based on “screening” [70]. In that study, the use of eccentric hamstring strength, age, and previous hamstring strain injury [70] was not able to predict the risk of injury. In this latter study, the Bayesian network was applied in professional Australian football players with the area under the curve (AUC) of 54% being classified as a poor performance metric [70]. On the other hand, when the artificial neural network based on “training load” was used with a sample of professional athletes of the same sport, it was recommended by the authors [67]. The prediction of performance in Australian football was based on heart rate [38] and “technical and tactical analysis” [39, 40] data with the use of decision tree classifier (*n* = 3 studies in total). The authors concluded that AI could add benefits and, hence, was recommended in Australian football [38–40].

Injuries cost over 1 billion dollars to Major League Baseball teams, and thus, preventing them is of high priority in that sport too [95]. There is a predominance of upper extremity injuries in baseball players, the surgical procedure most closely associated with baseball being the reconstruction of the ulnar collateral ligament of the elbow [96]. So far, AI has been used to predict the injury risk after ulnar collateral ligament reconstruction in professional baseball players [76]. With regard to performance improvement in baseball [95], we only found AI approaches in the “technical and tactical analysis” area [32, 37, 57, 58]. The AI techniques or methods used to predict sporting performance (*n* = 4 studies) in professional baseball players were support vector machine [57, 58], artificial neural network [32], and Bayesian network [37].

Data show that American football players who sustained concussions may experience significant salary reductions and perform worse after concussion [97]. According to Navarro et al. [97], the year-over-year change in contract value for the concussion group resulted in a mean overall salary reduction of USD $300 k ± $1,300 k/year. Moreover, the performance score reduction for all offensive scoring players sustaining concussions was statistically significant (i.e., pre, 9 ± 6 fantasy football point “FFP”s/game; post, 7 ± 4 FFPs/game). These aforementioned factors supported the search for AI techniques and methods to assist in predicting the risk of a concussion in a more accurate way [74, 75]. In addition, AI techniques or methods on “technical and tactical analysis” area have been applied in American football players [46, 54]. This sample was composed by collegiate [54, 75], professional, [46] and retired professional [74]. The most frequently used methods in that sport were the support vector machine (50%) [74, 75] and decision tree classifier (25%) [46] with Markov process (25%) [54].

With regard to the rest of team sports, AI applications were reported in professional ice hockey (Markov process [56] and support vector machine [59, 60]), cricket (*K*-means clustering [49]), field hockey (decision tree classifier [47]), and rugby (artificial neural network [34]). All these AI applications were on the “technical and tactical analysis” area, confirming the importance of these aspects for success in the team sports [98]. With regard to injury risk prediction, the decision tree classifier has been used in professional rugby players using the “training load” as the predictor [72]. In beach volleyball players, the monitoring data from wearable sensors has been used to model the injury risk via artificial neural network [68]. These studies highlight the importance of adequate management of training load in these sports too [99–101] along with the use of technology and innovative approaches in data management in order to protect the athletes’ health [102, 103].

### Limitations

This article presents the AI techniques or methods mostly used to predict injury risk and sporting performance in team sport athletes from research published in peer-reviewed journals in the last 5 years. Whether the same techniques and methods can be applied in individual sports remains an unanswered question. In the manuscripts were found differences on sample sizes, where some samples were not as large as others. However, this may have been a consequence of the large number of elite athletes who were part of the pooled sample, where 76% of them were professional athletes and for some sports it is not as common to obtain large samples of professional athletes as others.

## Conclusions

Our analysis showed that the AI techniques or methods for predicting injury risk and sporting performance mostly used in team sports were artificial neural networks, decision tree classifier, support vector machine, and Markov process. The team sports with the most AI applications were soccer, basketball, handball, and volleyball. The current state of development in the area proposes a promising future with regard to AI use in team sports. Further evaluation research based on prospective methods is warranted to establish the predictive performance of specific AI techniques and methods.

## Additional Files

Additional file 1: **Table S1. **Risk of bias score. (PDF 545 kb)

Additional file 2: **Table S2. **Summary of the AI studies. (PDF 662 kb)

## Data Availability

After publication, all data necessary to understand and assess the conclusions of the manuscript are available to any reader of Sports Medicine-Open.

## Abbreviations

AI: Artificial intelligence
FFP: Fantasy football point
NCAA: National Collegiate Athletic Association
PRISMA: Preferred Reporting Items for Systematic Reviews and Meta-Analyses
R&D: Research and Development
