# Supplementary material for: Current Approaches to the Use of Artificial Intelligence for Injury Risk Assessment and Performance Prediction in Team Sports: a Systematic Review
Source: Sports Med Open. 2019 Jul 3;5:28. doi: 10.1186/s40798-019-0202-3 (PMC6609928; doi:10.1186/s40798-019-0202-3)
Supplement: Supplementary file 1 — Table S1. Risk of bias score. (PDF 545 kb) [file 40798_2019_202_MOESM1_ESM.pdf]

Sports Medicine – Open. Current approaches to the use of artificial intelligence for injury risk assessment and performance prediction in team sports: a systematic review. João Gustavo Claudino, Daniel de Oliveira Capanema, Thiago Vieira de Souza, Julio Cerca Serrão, Adriano C. Machado Pereira, George P. Nassis. Corresponding author: João Gustavo Claudino; claudinojgo@usp.br. University of São Paulo, School of Physical Education and Sport - Laboratory of Biomechanics. Av. Prof. Mello de Morais, 65 – Cidade Universitária. Postal Code: 05508-030, São Paulo, São Paulo, Brazil.

	A	B	C	D	E	∑
2016 Abdullah [30]	1	1	2	2	1	7
2017 Adetiba [64]	1	1	2	2	1	7
2017 Bartlett [67]	1	1	2	2	1	7
2017 Bianchi [25]	1	1	1	2	1	6
2017 Bock [46]	1	1	1	2	1	6
2015 Bock [57]	1	1	1	2	1	6
2016 Carpita [45]	1	1	1	2	1	6
2018 Cene [43]	1	1	1	2	1	6
2015 Croft [34]	1	1	1	2	1	6
2015 Demers [59]	1	1	1	2	1	6
2018 Ertelt [65]	1	1	2	2	1	7
2016 Fuster-Parra [35]	1	1	2	2	1	7
2017 Ge [66]	1	1	1	2	1	6
2016 Goswami [74]	1	1	2	2	1	7
2016 Gu [60]	1	1	1	2	1	6
2017 Haiyan [53]	1	1	1	2	1	6
2017 Hassan [27]	1	1	2	2	1	7
2017 Hassan [28]	1	1	1	2	1	6
2017 Healey [37]	1	1	1	2	1	6
2017 Hoch [48]	1	1	1	2	1	6
2017 Jaspers [73]	1	1	2	2	1	7
2014 Jelinek [38]	1	1	1	2	1	6
2017 Kautz [68]	1	1	1	2	1	6
2015 Kempe [24]	1	1	1	2	1	6
2017 Kolbush [54]	1	1	1	2	1	6
2017 Leicht [41]	1	1	1	2	1	6
2017 Leicht [42]	1	1	1	2	1	6

	A		
**Criteria**	Peer reviewed		
**Definition**	Study published in peer-reviewed journal		
**Scoring**	0<br>No	1<br>Yes	2<br>-
	B		
**Criteria**	Real-world approach		
**Definition**	The approach was performed with real results/data of the athletes		
**Scoring**	0<br>No	1<br>Yes	2<br>-
	C		
**Criteria**	Population defined		
**Definition**	Age, gender, sport, level were described		
**Scoring**	0<br>No	1<br>Partly	2<br>Yes
	D		
**Criteria**	Experimental design		
**Definition**	Experimental design the study period was described and replicable		
**Scoring**	0<br>No	1<br>Partly	2<br>Yes
	E		
**Criteria**	Artificial Intelligence		

2013 Li [20]	1	1	1	2	1	6
2017 Link [36]	1	1	1	2	1	6
2017 Liu [a]	1	0	0	2	1	4
2018 López-Valenciano [15]	1	1	1	2	1	6
2013 Lu [21]	1	1	1	2	1	6
2015 Montoliu [44]	1	1	1	2	1	6
2013 Morgan [45]	1	1	1	2	1	6
2017 Pai [62]	1	1	1	2	1	6
2017 Park [32]	1	1	1	2	1	6
2018 Pensgaard [69]	1	1	2	2	1	7
2016 Qilin [63]	1	1	1	2	1	6
2016 Robertson [39]	1	1	1	2	1	6
2018 Ruddy [70]	1	1	2	2	1	7
2014 Sankaran [49]	1	1	1	2	1	6
2017 Schrapf [29]	1	1	1	2	1	6
2017 Schulte [56]	1	1	1	2	1	6
2013 Sheng [52]	1	1	1	2	1	6
2016 Soto Valero [58]	1	1	1	2	1	6
2017 Strnad [31]	1	1	1	2	1	6
2017 Sui [b]	1	0	0	2	1	4
2017 Thornton [72]	1	1	1	2	1	6
2015 Tilp [26]	1	1	1	2	1	6
2017 Tümer [33]	1	1	1	2	1	6
2015 Vales-Alonso [51]	1	1	1	2	1	6
2014 Wang [50]	1	1	1	2	1	6
2018 Wang [61]	1	1	2	2	1	7
2017 Wang [c]	1	0	0	2	1	4
2017 Wen [d]	1	0	0	2	1	4
2016 Whiteside [76]	1	1	2	2	1	7
2018 Woods [40]	1	1	2	2	1	7
2013 Wu [22]	1	1	1	2	1	6

**Definition**	The Artificial Intelligence approaches/techniques were described		
**Scoring**	0<br>No	1<br>Yes	2<br>-

2018 Wu [75]	1	1	1	2	1	6
2013 Xing [e]	1	0	0	2	1	4
2014 Zhang [23]	1	1	1	2	1	6
2017 Zi [55]	1	1	1	2	1	6
2018 Rossi [71]	1	1	2	2	1	7

Additional file 1: Table S1. Risk of bias score.

∑ = sum

a) Liu P, Wang Y. Research on College Basketball Tactics based on Data Mining Clustering Technology. Rev Facul Ing U.C.V., 32(12), 417-422, 2017.

b) Sui G. Research on competition tactical analysis in basketball teaching based on data mining technology. Agro Food Industry Hi-Tech, 28(3), 2584–2587, 2017.

c) Wang Z, Chen D. An optimized data mining algorithm application in volleyball match technique and competition tactics analysis. Tech Bul, 782–787, 2017.

d) Wen B. Technical analysis of a basketball match based on data mining. Agro Food Industry Hi-Tech, 28(1), 1953–1957, 2017.

e) Xing J, Zheng X, Zhang S, et al. Computer simulation evaluation model of basketball match based on fuzzy neural network. BioTechnology, 8(7), 910–915, 2013.
