# Supplementary material for: Current Approaches to the Use of Artificial Intelligence for Injury Risk Assessment and Performance Prediction in Team Sports: a Systematic Review
Source: Sports Med Open. 2019 Jul 3;5:28. doi: 10.1186/s40798-019-0202-3 (PMC6609928; doi:10.1186/s40798-019-0202-3)
Supplement: Supplementary file 2 — Table S2. Summary of the AI studies. (PDF 662 kb) [file 40798_2019_202_MOESM2_ESM.pdf]

Sports Medicine – Open. Current approaches to the use of artificial intelligence for injury risk assessment and performance prediction in team sports: a systematic review. João Gustavo Claudino, Daniel de Oliveira Capanema, Thiago Vieira de Souza, Julio Cerca Serrão, Adriano C. Machado Pereira, George P. Nassis. Corresponding author: João Gustavo Claudino; claudinojgo@usp.br. University of São Paulo, School of Physical Education and Sport - Laboratory of Biomechanics. Av. Prof. Mello de Morais, 65 – Cidade Universitária. Postal Code: 05508-030, São Paulo, São Paulo, Brazil.

Article (1st author)	Aspects (Type)	Sample (Profile; n)	Main Method	Complete Method	Model Evaluation Metrics
2013 Li [20]	Performance (Technical and Tactical Analysis)	Basketball (Professional, n = nr, males)	Artificial Neural Network	Artificial Neural Network with Back Propagation	Percentage of relative error = 0.08%; Mean square error = 0.0001
2013 Lu [21]	Performance (Physical Technical and Technical Analysis)	Basketball (Youth Academy, n = nr, males)	Artificial Neural Network	Fuzzy Clustering + Technique for Order of Preference by Similarity to Ideal Solution + Radial Basis Function Neural Network	Model Error = 4.96062e-007 (very small)
2013 Wu [22]	Performance (Technical and Tactical Analysis)	Basketball (Professional, n = nr, females)	Artificial Neural Network	Artificial Neural Network with Back Propagation + Fuzzy Comprehensive Evaluation	Not reported
2014 Zhang [23]	Performance (Technical and Tactical Analysis)	Basketball (Professional, n = nr, males)	Artificial Neural Network	Radial Basis Function	Prediction = 85%
2015 Kemp [24]	Performance (Technical and Tactical Analysis)	Basketball (Semi-Professional, n = 10, males)	Artificial Neural Network	Self-Organizing Maps	Accuracy = 98%
2017 Bianchi [25]	Performance (Technical and Tactical Analysis)	Basketball (Professional; n = 476, males)	Artificial Neural Network	Fuzzy Clustering Algorithm + Polynomial Fuzzifier Function + Multidimensional Scaling + Self-Organizing Map (Artificial Neural Network)	Not reported
2015 Tilp [26]	Performance (Technical and Tactical Analysis)	Handball (National Team U18, n = nr, males)	Artificial Neural Network	Artificial Neural Network	Not reported
2017 Hassan [27]	Performance (Technical and Tactical Analysis)	Handball (Youth Academy U18; n = 14, males)	Artificial Neural Network	Self Organizing Maps (Artificial Neural Network)	Mean distance = 0.49 ± 0.20 m (high similarity)

2017 Hassan [28]	Performance (Technical and Tactical Analysis)	Handball (National Teams U18; n = nr, males)	Artificial Neural Network	Radial Basis Function + Gaussian Classifier + Gradient Descent Algorithm	Mean distance = 1.20 ± 0.46) m (high accuracy); RMSE = 0.52 m (x direction) and 0.32 m (y direction)
2017 Schrapf [29]	Performance (Technical and Tactical Analysis)	Handball (National Teams U18; n = nr, males)	Artificial Neural Network	Artificial Neural Network	Not reported
2016 Abdullah [30]	Performance (Technical and Tactical Analysis)	Soccer (Youth Academy U17; n = 184, males)	Artificial Neural Network	Principal Component Analysis + Hierarchical Agglomerative Cluster Analysis + Artificial Neural Network with Back Propagation	R^2 = 92%; RMSE = 0.190; MR = 0.065
2017 Strnad [31]	Performance (Match Attendance)	Soccer (Professional, n = nr, males)	Artificial Neural Network	Logistic transfer function + Multi Layer Perceptron + Three-Layer Feedforward Neural Network + Epoch Wise Back Propagation	MAPE = 3.68% ± 0.03%; MAE = 785 ± 7
2017 Park [32]	Performance (Technical and Tactical Analysis)	Baseball (Professional, n = nr, males)	Artificial Neural Network	Adam optimization technique + Deep Neural network	GM = 79%; Sensitivity = 98%; Specificity = 63%
2017 Tümer [33]	Performance (Technical and Tactical Analysis)	Volleyball (Professional, n = nr, males)	Artificial Neural Network	Multi-Layer Perceptron with Levenberg – Marquardt Back Propagation + Logsig Function	Accuracy = 98%; MSE = 0.005337
2015 Croft [34]	Performance (Technical and Tactical Analysis)	Rugby (Professional; n = nr, males)	Artificial Neural Network	Self-Organizing Map	Qualitative Analysis (highlight colors)
2016 Fuster-Parra [35]	Performance (Psychological Dynamics of Cooperative Team Work)	Soccer (Semi-Professional; n = 403, males)	Bayesian Network	Global Markov property + Bayesian network	Accuracy = 90% ± 0.3%; Sensitivity = 92 ± 0.2%; Specificity = 65% ± 0.4%; Precision = 98% ± 0.3%
2017 Link [36]	Performance (Technical and	Soccer (Professional, n = nr,	Bayesian Network	Rauch-Tung-Striebel + Bayesian Network	Recall = 84%; Precision = 92%; F-Score = 87%

	Tactical Analysis)	males)			
2017 Healey [37]	Performance (Technical and Tactical Analysis)	Baseball (Professional; n = nr, males)	Bayesian Network	Bayes theorem + Kernel Methods + Cross-Validation + Weighted on Base Average	Not reported
2014 Jelinek [38]	Performance (Prediction based on Heart Rate)	Australian Football (Professional; n = 40, males)	Decision Tree Classifier	Random Forest + Meta Regressions	R^2 = 74%
2016 Robertson [39]	Performance (Technical and Tactical Analysis)	Australian Football (Professional, n = nr, males)	Decision Tree Classifier	One-way ANOVA + Logistic Regression + Classification Trees	Accuracy = 90%
2018 Woods [40]	Performance (Technical and Tactical Analysis)	Australian Football (Academy U18, n = 244, males)	Decision Tree Classifier	PART decision list	Accuracy = 70%
2017 Leicht [41]	Performance (Technical and Tactical Analysis)	Basketball (Professional, n = nr, males)	Decision Tree Classifier	Spearman's Correlation Matrix + MANOVA + Cohen's d Statistic + Conditional Interference + Classification Tree	Accuracy = 81%
2017 Leicht [42]	Performance (Technical and Tactical Analysis)	Basketball (Professional, n = nr, females)	Decision Tree Classifier	MANOVA + Cohen's d Statistic + Conditional Interference + Classification Tree	Accuracy = 86%
2018 Cene [43]	Performance (Technical and Tactical Analysis)	Basketball (Professional; n = nr, males)	Decision Tree Classifier	K-means Cluster Analysis + Bayesian Model Averaging + Classification Tree	Accuracy = 80%
2015 Montoliu [44]	Performance (Technical and Tactical Analysis)	Soccer (Professional, n = nr, males)	Decision Tree Classifier	Bag-of-Words + Random Forest	Accuracy = 93% ± 0.2%
2016 Carpita [45]	Performance (Technical and Tactical Analysis)	Soccer (Professional, n = 746, males)	Decision Tree Classifier	Random Forests + Principal Component Analysis + Multinomial Logistic Regression	Accuracy = 64%; Specificity = 69%; Sensitivity = 84%
2017 Bock [46]	Performance (Technical and	American Football (Professional; n = nr,	Decision Tree Classifier	Bernoulli Distribution Loss Function + Logistic Regression + Gradient	False discovery rates = < 15%.

	Tactical Analysis)	males)		Boosting Machines	
2013 Morgan [47]	Performance (Technical and Tactical Analysis)	Field Hockey (Professional, n = 8, females)	Decision Tree Classifier	Decision Tree Induction	Accuracy = 64%; AUC = 71%
2017 Hoch [48]	Performance (Technical and Tactical Analysis)	Soccer (Professional; n = 20, males)	Fuzzy Clustering	Fuzzy Rule Bases + Laplacian Score + Spectral Clustering Method + Fuzzy Clustering	Not reported
2014 Sankaran [49]	Performance (Technical and Tactical Analysis)	Cricket (Professional, n = 104, males)	K-Means Clustering	K-Means Cluster Analysis	Not Reported
2014 Wang [50]	Performance (Technical and Tactical Analysis)	Soccer (Professional, n = nr, males)	K-Means Clustering	Genetic Algorithm + Self Organizing Map	Possession percentage = 57%; Free kick 17%
2015 Vales-Alonso [51]	Performance (Physical, Technical and Tactical Analysis)	Volleyball (Professional, n = nr, females)	K-Nearest Neighbour	Bellman’s equation + Markov decision process + k-NN Classifier	True positives = 93%; True negatives = 100%
2013 Sheng [52]	Performance (Technical and Tactical Analysis)	Volleyball (Professional, n = nr)	Markov Process	Fuzzy evaluation + Markov Process	Not reported
2017 Haiyan [53]	Performance (Technical and Tactical Analysis)	Volleyball (Professional; n = nr, females)	Markov Process	Random Sampling Techniques + Markov Process	Not reported
2017 Kolbush [54]	Performance (Technical and Tactical Analysis)	American Football (Collegiate, n = nr, males)	Markov Process	Logistic Regression + Markov Chain Model	Accuracy = 61%
2017 Zi [55]	Performance (Technical and Tactical Analysis)	Basketball (Professional, n = nr, males)	Markov Process	Apriori Algorithm + Markov Process	Not reported
2017 Schulte [56]	Performance (Technical and	Ice Hockey (Professional, n =	Markov Process	Affinity Propagation + Markov Game Formalism + Dynamic Programming	Average Goal Ratio, ρ = 0.7; Average Team Winning Chance = 82%

	Tactical Analysis)	2233, males)			
2015 Bock [57]	Performance (Technical and Tactical Analysis)	Baseball (Professional, n = 402, males)	Support Vector Machine	Cross-Validation + Support Vector Machine + Radial Basis Function Kernels	Accuracy = 70%; Predictability = 75%.
2016 Soto Valero [58]	Performance (Technical and Tactical Analysis)	Baseball (Professional, n = nr, males)	Support Vector Machine	Attribute ranking technique + Support Vector Machine + Sequential Minimal Optimization	Accuracy = 59%
2015 Demers [59]	Performance (Technical and Tactical Analysis)	Ice Hockey (Professional; n = nr, males)	Support Vector Machine	Sigmoid Function + Minimizing the Negative Log-Likelihood Function + Support Vector Machine	Percentage Correctly Predicted = 70%
2016 Gu [60]	Performance (Technical and Tactical Analysis)	Ice Hockey (Professional; n = nr, males)	Support Vector Machine	Wilcoxon Rank-Sum Test + Support Vector Machine + Analytic Network Process	Accuracy = 78%
2018 Wang [61]	Performance (Technical and Tactical Analysis)	Volleyball (Professional, n = 10, males)	Support Vector Machine	Principal Component Analysis + Support Vector Machine	Accuracy = 94%; Recall = 93%; F1-score = 93%
2017 Pai [62]	Performance (Technical and Tactical Analysis)	Basketball (Professional, n = nr, males)	Support Vector Machine + Decision Tree Classifier	Correlation-based Feature Selection + Support Vector Machine + Decision Tree Classifier	Accuracy = 85%
2016 Qilin [63]	Injury (Training Process/Knee Injury Causes)	Soccer (Collegiate, n = 60, males)	Artificial Neural Network	Exploratory Factor Analysis + Factory Analysis + Artificial Neural Network with Back Propagation	KMO = 87%
2017 Adetiba [64]	Injury (Heart Defect Detection)	Soccer and Basketball (Professional; n = 5, males and n = 5, females)	Artificial Neural Network	Fast Fourier Transform + Inverse Fourier Transform + Multi-Layer Perceptron-Artificial Neural Network based Back Propagation + Multi-Layer Perceptron - Artificial Neural Network based Levenberg-Marquardt + Tangent Sigmoid Activation(hidden and output)	Accuracy = 98%; Sensitivity, = 98%; Specificity = 99%; MSE = 0.0093

2018 Ertelt [65]	Injury (Ground Reaction Force Pattern)	Soccer, Volleyball and Handball (Professional; n = ~9, males)	Artificial Neural Network	Normalizations and Approximations + Bayesian Regularization Artificial Neural Network	Accuracy = 94%; Predictability = 94%
2017 Ge [66]	Injury (Training Process/Knee Injury Causes)	Basketball (Collegiate; n = nr, males)	Artificial Neural Network	Exploratory Factor Analysis + Factory Analysis + Artificial Neural Network with Back Propagation	KMO = 87%
2017 Bartlett [67]	Injury (Training load)	Australian Football (Professional; n = 41, males)	Artificial Neural Network	Generalised Estimating Equations + Artificial Neural Network (Individually)	RMSE = 1.24 ± 0.41
2017 Kautz [68]	Injury (Monitoring based on Wearable Sensors)	Beach Volleyball (Youth Academy - Professional; n = 11, females and n = 19, males)	Artificial Neural Network	Decision Tree (C4.5) + Deep Convolutional Neural Network	Accuracy = 83%
2018 Pensgaard [69]	Injury (Psychosocial Stress Factors)	Soccer (Professional, n = 193, females)	Bayesian Logistic Regression	Bayesian Correlation Analyses + Bayesian Logistic Regression + Markov Chain Monte Carlo	Not Reported
2018 Ruddy [70]	Injury (Screening)	Australian Football (Professional, n = 362, males)	Bayesian Network	Synthetic Minority Oversampling Techniques + Naive Bayes	AUC = 54%
2018 López-Valenciano [15]	Injury (Screening)	Soccer and Handball (Professional, n = 132, males)	Decision Tree Classifier	Synthetic Minority Oversampling Technique + Bagging-based Cost-Sensitive Classifier + Adtree	AUC = 75%; True Positive Rate = 66%; True Negative Rate = 79%
2018 Rossi [71]	Injury (Training Load)	Soccer (Professional, n = 26, males)	Decision Tree Classifier	Cross Validation + Adaptive Synthetic Sampling Approach + Decision Tree Classifier	Recall = 80% ± 0.07; Precision = 50% ± 0.11; F1-score = 64% ± 0.10; AUC = 76% ± 0.12
2017 Thornton [72]	Injury (Training Load)	Rugby (Professional, n = 25, males)	Decision Tree Classifier	Generalized Estimating Equations + Random Forest	ROC = 64% ± 0.04

2018 Jaspers [73]	Injury (Training Load)	Soccer (Professional; n = 38, males)	Least Absolute Shrinkage and Selection Operator	Naive Baseline Method + Least Absolute Shrinkage and Selection Operator (For Group)	MAE = 0.80 (0.78 – 0.82)
2016 Goswami [74]	Injury (Concussion)	American Football (Retired Professional; n = 19, males)	Support Vector Machine	Cross-validation (Leave-One-Out), Radial Basis Function + Logistic Regression Classifier (Elasticnet + Glmnet + LRC + ADMM)	Accuracy = 83%; AUC = 81%
2018 Wu [75]	Injury (Concussion)	American Football (Collegiate, n = 419, males)	Support Vector Machine	Principal Component Analysis + Support Vector Machine	Accuracy = 94%; Precision = 94%; Sensitivity = 94%; Specificity = 94%
2016 Whiteside [76]	Injury (Ulnar Collateral Ligament Reconstruction)	Baseball (Professional, n = 208, males)	Support Vector Machine	Logistic Regression + Support Vector Machine	Accuracy = 75%

Additional file 2: Table S2. Summary of the AI studies.

n = sample size; nr = not reported; AUC = area under the curve; R^2 = Correlation of Determination; RMSE = Root Mean Square Error; MR = Misclassification Rate; MSE = Mean Squared Error; MAE = Mean Absolute Error; GM = Geometric mean of sensitivity and specificity to consider the positive and negative classes equally; nr = not reported; KMO = Kaiser-Meyer-Olkin Test; ROC = Receiver Operating Characteristic curves, providing information regarding the sensitivity (true positive rate) by one minus specificity (false positive rate).
